# Calculation of Energy Diagram of Asymmetric Graded-Band-Gap Semiconductor Superlattices

**DOI:** 10.1186/s11671-017-1981-4

**Published:** 2017-03-20

**Authors:** Liubomyr S. Monastyrskii, Bogdan S. Sokolovskii, Mariya P. Alekseichyk

**Affiliations:** Department of Radioelectronic and Computer Systems, Faculty of Electronics and Computer Technologies, Ivan Franko National University of Lviv, 50 Dragomanov Street, 79005 Lviv, Ukraine

**Keywords:** Graded-band-gap semiconductors, Energy diagram, Superlattices, Multilayer structures, 73.20.–r, 71.20.–b, 73.21.Ac, 73.40.Lq

## Abstract

The paper theoretically investigates the peculiarities of energy diagram of asymmetric graded-band-gap superlattices with linear coordinate dependences of band gap and electron affinity. For calculating the energy diagram of asymmetric graded-band-gap superlattices, linearized Poisson’s equation has been solved for the two layers forming a period of the superlattice. The obtained coordinate dependences of edges of the conduction and valence bands demonstrate substantial transformation of the shape of the energy diagram at changing the period of the lattice and the ratio of width of the adjacent layers. The most marked changes in the energy diagram take place when the period of lattice is comparable with the Debye screening length. In the case when the lattice period is much smaller that the Debye screening length, the energy diagram has the shape of a sawtooth-like pattern.

## Background

The graded-band-gap semiconductors have attracted the attention of scientists since the year 1957 when H. Kroemer puts forward the idea about quasielectric and quasimagnetic fields [1] which, in contrast with the conventional fields, act in a different way upon electrons and holes. The presence of such fields is a unique feature of semiconductors with spatially nonhomogeneous composition that leads to formation in these semiconductors in a number of properties [2] which are of interest for many practical applications, particularly for fabrication of efficient solar cells [3, 4]. The strength of the quasielectric field is proportional to the gradient of composition, and achieving its large and constant value is possible at small thickness of specimens. The thickness of structures can be increased without decreasing the quasielectric field intensity when one uses multilayer structures or superlattices. In such structures, it is possible to observe in the pronounced form of the manifestations of the quasielectric field. Since the properties of graded-band-gap structures strongly depend on the shape of the energy band diagram, ascertainment of its peculiarities is the necessary and first stage at studying these structures. Firstly, the features of formation of the energy diagram of graded-band-gap superlattices were established in [5, 6] for the case of the symmetric form of the latter. These superlattices belong to those of a classical type [7–9] in which the superlattice’s period is much greater than the de Broglie wavelength and therefore quantization of the energy spectra of electrons and holes does not take place. The aim of this research is to theoretically investigate the peculiarities of energy diagram of classical asymmetric graded-band-gap superlattices with linear coordinate dependences of band gap and electron affinity.

## Methods

Constructing an energy band diagram means to plot coordinate dependences of conduction band bottom *E*_c_ and valence band ceiling *E*_v_ which are reckoned from the vacuum level *E*_0_ and expressed through the electrostatic energy$\hbox{---} e\varphi,$ the electron affinity χ and the band gap *E*_g_:

1 $$ {E}_c(x)={E}_0- e\varphi (x)-\chi (x), $$

**Fig. 1:**
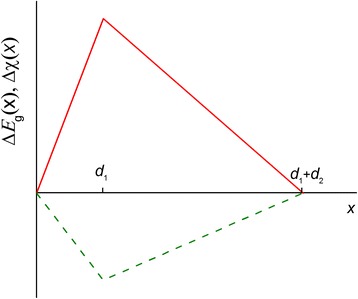
Schematic of coordinate profiles of band gap (*solid line*) and electron affinity (*dashed line*) within one period of the superlattice

In this work, we will consider the sawtooth-like type of graded-band-gap superlattice in which *E*_g_ and *χ* are piecewise linear functions (Fig. 1).

The coordinate dependence of electrostatic potential *φ* can be found from Poisson’s equation

2 $$ \varepsilon {\varepsilon}_0\frac{d^2\varphi}{d{ x}^2}= e\left[{N}_{\mathrm{c}} \exp \left(\frac{E_{\mathrm{F}}-{E}_{\mathrm{c}}(x)}{kT}\right)-{N}_{\mathrm{v}} \exp \left(\frac{E_{\mathrm{v}}(x)-{E}_{\mathrm{F}}}{kT}\right)+{N}_{\mathrm{a}}-{N}_{\mathrm{d}}\right]. $$

In Eq. (2), written for the case of carrier nondegeneracy, *ε* and *ε*_0_ represent the dielectric constants of the material and free space; *N*_c_ and *N*_v_ are the effective densities of states in the conduction and valence bands, *N*_a_ and *N*_d_ are the concentrations of acceptors and donors, and *E*_F_ is the constant Fermi level of the structure.

The electron, *n*, and hole, *p*, concentrations appearing in Eq. (2) can be written as follows:

3 $$ n(x)={N}_{\mathrm{c}} \exp \left[\frac{\zeta (x)}{kT}\right]={n}_0(0) \exp \left[\frac{e\varphi +\varDelta \chi (x)}{kT}\right], $$

4 $$ p(x)={N}_{\mathrm{v}} \exp \left[-\frac{E_{\mathrm{g}}(x)+\zeta (x)}{kT}\right]={p}_0(0) \exp \left[-\frac{\varDelta {E}_{\mathrm{g}}(x)+\varDelta \chi (x)+ e\varphi (x)}{kT}\right], $$

where ζ_0_(*x*) = *E*_F_ − *E*_c_(*x*), Δ*E*_g_(*x*) = *E*_g_(*x*) − *E*_g_(0), Δ*χ*(*x*) = Δ*χ*(*x*) – Δ*χ*(0), *n*_0_(0), and *p*_0_(0) are respectively the concentrations of electron and holes in an uniform semiconductor with the same composition (band gap) and doping level as in the graded-band-gap multilayer structure at the point *x* = 0. From the neutrality equation, we have

5 $$ {n}_0(0)={N}_{\mathrm{c}} \exp \left[\frac{\zeta_0(0)}{kT}\right], $$

6 $$ {p}_0(0)={N}_{\mathrm{v}} \exp \left[-\frac{E_{\mathrm{g}}(0)+{\zeta}_0(0)}{kT}\right]=\frac{n_i^2(0)}{n_0(0)}, $$

where *ζ*_0_(0) is the value of function

7 $$ {\zeta}_0(x)= k T \ln \left[\frac{N_{\mathrm{d}}-{N}_{\mathrm{a}}+\sqrt{{\left({N}_{\mathrm{d}}-{N}_{\mathrm{a}}\right)}^2+4{n}_i^2(x)}}{2{N}_{\mathrm{c}}}\right] $$

in the point *x* = 0. Here, function *ζ*_0_(*x*) has the meaning of difference between the Fermi level and conduction band bottom (i.e., chemical potential of electrons) in the uniform semiconductor with the parameters corresponding to the point *x* of our structure.

For the profiles of *E*_g_(*x*) and *χ*(*x*) presented in Fig. 1, Poisson’s equation can be written in the following dimensionless form:

8 $$ \frac{d^2\overline{\varphi}}{d{\xi}^2}=\left(1-\kappa \right) \exp \left(\overline{\varphi}+\beta \xi \right)-\kappa \exp \left[-\overline{\varphi}-\left(\delta +\beta \right)\xi \right]+\overline{N} $$

at $$ 0\le \xi <{\overline{d}}_1 $$ and

9 $$ \frac{d^2\overline{\varphi}}{d{\xi}^2}=\left(1-\kappa \right) \exp \left[\overline{\varphi}-\beta \left[\nu \left(\xi -{\overline{d}}_1\right)+{\overline{d}}_1\right]\right]-\kappa \exp \left[-\overline{\varphi}+\left(\delta +\beta \right)\left[\nu \xi -\left(\nu +1\Big){\overline{d}}_1\right)\right]\right]+\overline{N} $$

at $$ {\overline{d}}_1\le \xi <{\overline{d}}_1+{\overline{d}}_2, $$

where $\overline{\varphi}= e\varphi / k T$, *ξ* = *x*/*L*_D_, ${L}_{\mathrm{D}}=\sqrt{\varepsilon {\varepsilon}_0 kT/{e}^2\left[{n}_0(0)+{p}_0(0)\right]}$, *δ* = (*L*_D_/*kT*)*dE*_g_/*dx* (at *x* < *d*_1_), *β* = (*L*_D_/*kT*)*dχ*/*dx* (at *x* < *d*_1_), *κ* = *p*_0_(0)/[*n*_0_(0) + *p*_0_(0)], *ν* = *d*_1_/*d*_2_, and $\overline{N}=\left({N}_{\mathrm{a}}-{N}_{\mathrm{d}}\right)/\left[{n}_0(0)+{p}_0(0)\right].$

Equations (8) and (9) should obey the following boundary conditions:

10 $$ \overline{\varphi}\left(\xi =-{\overline{d}}_1\right)=\overline{\varphi}\left(\xi =+{\overline{d}}_1\right),\overline{\varphi}\left(\xi =+0\right)=\overline{\varphi}\left(\xi =-{\overline{d}}_2\right), $$

11 $$ \frac{d\overline{\varphi}}{ d\xi}\left(\xi =-{\overline{d}}_1\right)=\frac{d\overline{\varphi}}{ d\xi}\left(\xi =+{\overline{d}}_1\right),\frac{d\overline{\varphi}}{ d\xi}\left(\xi =+0\right)=\frac{d\overline{\varphi}}{ d\xi}\left(\xi =-{\overline{d}}_2\right), $$

which reflect the continuity of electrostatic potential and electric field strength at the interfaces.

## Results and Discussion

For obtaining the analytical solution of boundary problem (8)–(11), we consider the case when the drops in *E*_g_ and *χ* are small in comparison with *kT*(|Δ*E*_g_(*d*_1_)|<<*kT*, |Δ*χ*(*d*_1_)|<<*kT*). Then, the right hand side of (8) and (9) can be linearized what allows us to obtain the following expressions for *E*_c_(ξ):

12 $$ \frac{E_{\mathrm{c}}\left(\xi \right)-{E}_{\mathrm{c}}(0)}{kT}=\kappa \delta \xi -\left(\nu +1\right)\left(\beta +\kappa \delta \right)\frac{ \sinh \left(\frac{{\overline{d}}_1}{2}\right) \sinh \left(\xi -\frac{{\overrightarrow{d}}_1}{2}\right)}{ \sinh \left(\frac{{\overline{d}}_1+{\overline{d}}_2}{2}\right)} $$

at $$ 0\le \xi <{\overline{d}}_1, $$

13 $$ \frac{E_{\mathrm{c}}\left(\xi \right)-{E}_{\mathrm{c}}(0)}{kT}=-\nu \kappa \delta \xi \kern0.34em +\left(\nu +1\right)\left(\beta +\kappa \delta \right)\kern0.5em \left[{\overline{d}}_1-\frac{ \exp \kern0.5em \left(\frac{{\overline{d}}_1}{2}\right)\kern0.5em  \sinh \kern0.5em \left(\frac{{\overline{d}}_1}{2}\right) \cosh \kern0.5em \left(\xi -\frac{{\overrightarrow{d}}_1+{\overline{d}}_2}{2}\right)}{ \sinh \kern0.5em \left(\frac{{\overline{d}}_1+{\overline{d}}_2}{2}\right)}\right] $$

at $$ {\overline{d}}_1\le \xi <{\overline{d}}_2+{\overline{d}}_1. $$

Since *E*_v_(ζ) = *E*_c_(ζ) − *E*_g_(ζ), the above expressions allow one to calculate the coordinate dependences of the valence band ceiling.

Let us analyze the general features of energy band diagram formation on the example of the simplified but quite realistic structure with intrinsic conductivity (*κ* = 0.5) in which the edge of valance band does not depend on the composition and therefore on the coordinate (*β* + *δ* = 0). Such a “common anion rule” [10] is fulfilled in a number of solid solutions on the basis of A_2_B_6_ and A_3_B_5_ compounds.

**Fig. 2:**
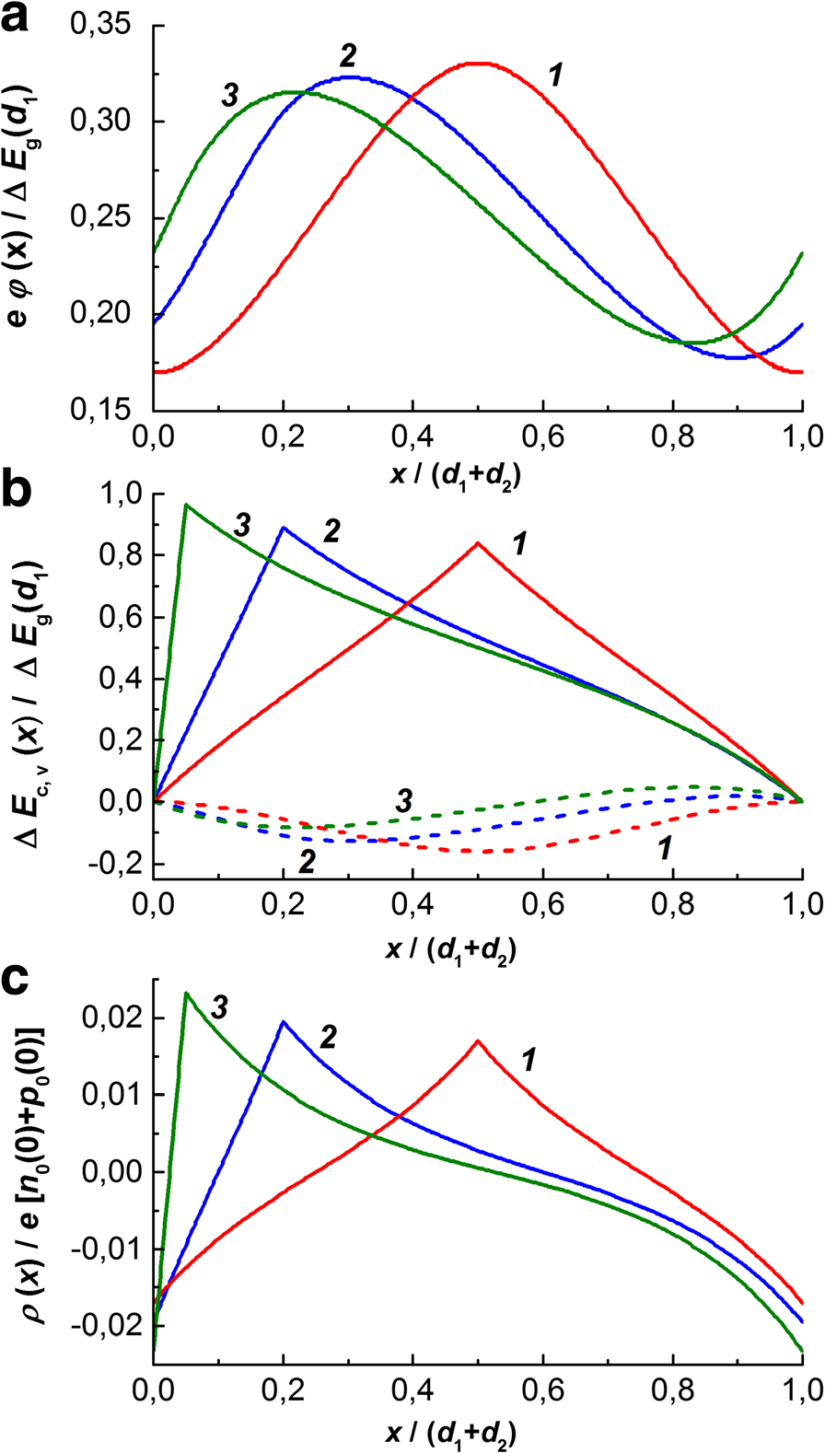
Coordinate dependences of electrostatic potential (**a**), edges of conduction band (*solid lines*) and valence band (*dashed lines*) (**b**), and charge density (**c**) for *d*_1_ + *d*_2_ = 5*L*_D_, Δ*E*_g_(*d*_1_) = 0.1*kT*, *κ* = 0.5, and *d*_1_/(*d*_1_ + *d*_2_) = 0.5 (*1*), 0.2 (*2*), and 0.05(*3*)

**Fig. 3:**
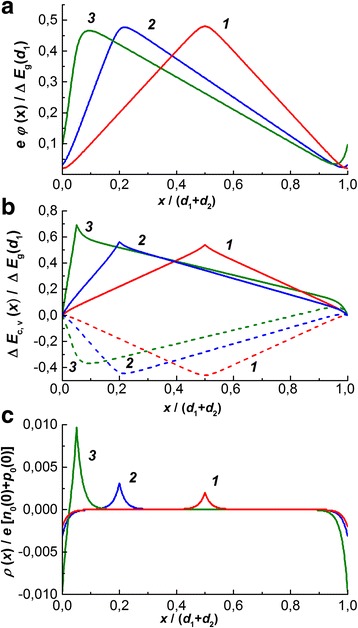
Coordinate dependences of electrostatic potential (**a**), edges of conduction band (*solid lines*) and valence band (*dashed lines*) (**b**), and charge density (**c**) for *d*_1_ + *d*_2_ = 50*L*_D_, Δ*E*_g_(*d*_1_) = 0.1*kT*, *κ* = 0.5, and *d*_1_/(*d*_1_ + *d*_2_) = 0.5 (*1*), 0.2 (*2*), and 0.05(*3*)

The calculated dependences are shown in Figs. 2 and 3 for the cases of comparable and large period with respect to the Debye screening length.

It follows from the solution of Eqs. (8) and (9) that contrary to the case of symmetric superlattices [5, 6], the electrostatic potential in the asymmetric ones nonmotonously depends on the coordinate within the layer of larger thickness (Figs. 2 and 3a) reaching there both minimum and maximum. These features are also manifested in the shape of the energy band diagram (Figs. 2 and 3b) especially in the case when the thickness of larger layer is of the order of the Debye length. Then, in two layers of the lattice’s period, the space charge is built up (Fig. 2c) with the integral electroneutrality being fulfilled within each layer. The charge of maximal density is located at the interfaces and its absolute value increases at increasing the degree of lattice asymmetry. When thickness of the lattice’s layer greatly exceeds the Debye length, the conduction and valence edges are characterized by the linear dependences in the whole volume for the exception of thin regions in the vicinity of interfaces (Fig. 3c).

For small values of the layer thickness (*d*_1_, *d*_2_<< *L*_D_), the electrostatic potential nearly does not depend on the coordinate

14 $$ \overline{\varphi}\left(\xi \right)\cong \frac{\beta +\kappa \delta}{2}. $$

Therefore,

15 $$ \varDelta {E}_{\mathrm{c}}\left(\xi \right)\cong \varDelta \chi \left(\xi \right), $$

16 $$ \varDelta {E}_{\mathrm{v}}\left(\xi \right)\cong \varDelta \chi \left(\xi \right)-\varDelta {E}_{\mathrm{g}}\left(\xi \right), $$

i.e., the profiles of the band edges are determined only by the coordinate dependences of *E*_g_ and *χ*. Such a property is also observed in doped superlattices.

## Conclusions

Charge carrier redistribution taking place in a sawtooth-like graded-band-gap superlattice leads to formation of energy band diagram which is characterized by the following features:

1. The shape of energy band diagram depends of the value of the superlattice’s period and the ratio of thicknesses of adjacent layers, with the most noticeable size dependence taking place when the superlattice’s period is of the order of the Debye length.
2. Contrary to the symmetric graded-band-gap superlattices, the extrema of conduction or valence band in the asymmetric superlattices are formed not at the interfaces but within the layer of larger thickness.
3. When the period of graded-band-gap superlattice is much smaller than the Debye length, the profiles of the band edges are determined exclusively by the coordinate dependences of band gap and electron affinity.
